# Prospective Role of Bioactive Molecules and Exosomes in the Therapeutic Potential of Camel Milk against Human Diseases: An Updated Perspective

**DOI:** 10.3390/life12070990

**Published:** 2022-07-04

**Authors:** Farheen Badrealam Khan, Mohammad Azam Ansari, Shahab Uddin, Abdul Rasheed Palakott, Irfa Anwar, Ahmad Almatroudi, Mohammad N. Alomary, Faris Alrumaihi, Faris F. Aba Alkhayl, Saad Alghamdi, Khalid Muhammad, Chih-Yang Huang, Jayasimha Rayalu Daddam, Haroon Khan, Sajid Maqsood, Mohammed Akli Ayoub

**Affiliations:** 1 Department of Biology, College of Science, The United Arab Emirates University, Al Ain 15551, United Arab Emirates; abdulrasheed1984@uaeu.ac.ae (A.R.P.); 202090095@uaeu.ac.ae (I.A.); k.muhammad@uaeu.ac.ae (K.M.); 2 Department of Epidemic Disease Research, Institutes for Research and Medical Consultations, Imam Abdulrahman Bin Faisal University, Dammam 31441, Saudi Arabia; 3 Translational Research Institute, Academic Health System, Hamad Medical Corporation, Doha 3050, Qatar; skhan34@hamad.qa; 4 Dermatology Institute, Academic Health System, Hamad Medical Corporation, Doha 3050, Qatar; 5 Laboratory of Animal Center, Qatar University, Doha 2731, Qatar; 6 Department of Medical Laboratories, College of Applied Medical Sciences, Qassim University, Qassim 51431, Saudi Arabia; aamtrody@qu.edu.sa (A.A.); f_alrumaihi@qu.edu.sa (F.A.); ffabaalkhiel@qu.edu.sa (F.F.A.A.); 7 National Centre for Biotechnology, King Abdulaziz City for Science and Technology (KACST), Riyadh 11442, Saudi Arabia; malomary@kacst.edu.sa; 8 Department of Pharmaceutical Chemistry and Pharmacognosy, College of Dentistry and Pharmacy, Buraydah Colleges, Buraydah 52571, Saudi Arabia; 9 Laboratory Medicine Department, Faculty of Applied Medical Sciences, Umm Al-Qura University, Makkah 21955, Saudi Arabia; ssalghamdi@uqu.edu.sa; 10 Department of Biotechnology, Asia University, Taichung 404, Taiwan; cyhuang@mail.cmu.edu.tw; 11 Graduate Institute of Biomedical Sciences, China Medical University, Taichung 404, Taiwan; 12 Cardiovascular and Mitochondrial Related Disease Research Center, Hualien Tzu Chi Hospital, Buddhist Tzu Chi Medical Foundation, Hualien 970, Taiwan; 13 Centre of General Education, Buddhist Tzu Chi Medical Foundation, Tzu Chi University of Science and Technology, Hualien 970, Taiwan; 14 Department of Medical Research, China Medical University Hospital, China Medical University, Taichung 404, Taiwan; 15 Department of Ruminant Science, Institute of Animal Sciences, Agriculture Research Organization, Volcani Center, Rishon Lezion 7505101, Israel; jayasimharayalu@gmail.com; 16 Department of Pharmacy, Abdul Wali Khan University Mardan, Mardan 23200, Pakistan; haroonkhan@awkum.edu.pk; 17 Department of Food Science, College of Agriculture and Veterinary Medicine, United Arab Emirates University, Al Ain 15551, United Arab Emirates; sajid.m@uaeu.ac.ae; 18 Zayed Center for Health Sciences, United Arab Emirates University, Al Ain 15551, United Arab Emirates

**Keywords:** bioactive peptides, camel milk, cancer, diabetes, molecular signaling, exosomes, human diseases, therapeutics

## Abstract

Camel milk (CM) constitutes an important dietary source in the hot and arid regions of the world. CM is a colloidal mixture of nutritional components (proteins, carbohydrates, lipids, vitamins, and minerals) and non-nutritional components (hormones, growth factors, cytokines, immunoglobulins, and exosomes). Although the majority of previous research has been focused on the nutritional components of CM; there has been immense interest in the non-nutritional components in the recent past. Reckoning with these, in this review, we have provided a glimpse of the recent trends in CM research endeavors and attempted to provide our perspective on the therapeutic efficacy of the nutritional and non-nutritional components of CM. Interestingly, with concerted efforts from the research fraternities, convincing evidence for the better understanding of the claimed traditional health benefits of CM can be foreseen with great enthusiasm and is indeed eagerly anticipated.

## 1. Introduction

It is widely recognized that camel milk (CM) is a valuable nutritional source for people living in the hot and arid regions of the world. CM is a complex biological fluid that contains not only nutritional components, including macronutrients and micronutrients, but also non-nutritional components, such as hormones, growth factors, immune system products, and exosomes [1,2,3].

Evidence has shown that CM is used for therapeutic benefits against various diseases and conditions [4,5,6,7,8,9]. To this end, CM has been reported to provide therapeutic benefits against various pathophysiological conditions, including diabetes, hypertension, cancer, inflammatory and allergic responses as discussed in subsequent sections [1,7,10,11,12,13]. Amongst all these beneficial properties, its therapeutic potential against diabetes has been extensively explored. Reports have shown that CM consumption not only lowers the prevalence of diabetes, but also improves the detrimental effects of hyperglycemic condition, as well as reducing the insulin therapy required by type-1 diabetic patients [5,6,7,11,14,15,16,17,18]. The antidiabetic potentials of CM were mainly accredited to the presence of insulin and/or insulin-like peptides in CM. However, this belief has undergone a paradigm shift as a consequence of independent studies that have explicitly highlighted the fact that that these insulin/insulin-like peptides are completely hydrolyzed by gastrointestinal enzymes [19,20,21] This has prompted the researcher to study the CM protein hydrolysates and their bioactive peptides; indeed, lately, this area of research has garnered much attention all across the globe [5,22,23,24].

Moreover, CM also possesses intriguing anti-microbial potentials, including antibacterial properties against both gram-negative and gram-positive bacteria, antifungal and anti-viral properties [9,13,25,26,27,28,29]. These beneficial anti-microbial effects were mainly attributed to the presence of greater amounts of protective proteins, as detailed in subsequent sections [9,25].

Furthermore, CM has been used for its therapeutic benefits against various other diseases, including jaundice, asthma, psoriasis, Crohn’s disease, autism and dropsy in various parts of the world [4,26,30,31].

It has to be acknowledged that in the past, several studies have focused on camel milk components, such as proteins, carbohydrates, and fatty acids [22,24,32,33,34]. Yet, in recent years, CM components that have not previously been well appreciated, such as extracellular vesicles (EVs), have also been actively researched, supported by technological advancements [1,35]. Reckoning with these aspects in mind, in this review, we discuss recent trends in CM research endeavors and provide a perspective on the therapeutic efficacy of the nutritional and non-nutritional components of CM.

## 2. Brief Overview of Camel Milk (CM) Components

### 2.1. Nutritional Components (Macronutrients and Micronutrients)

Over the years, much attention has been diverted toward the consumption of CM as an alternative to milk from other sources, apparently due to its therapeutic benefits and lower allergenicity issues. Indeed, the physicochemical composition of CM differs considerably from that of milk from other domestic dairy animals. It has been argued that the physicochemical composition of CM is dependent on various factors, including geographical origin, breed, age, parity, season, ecology, feed and feeding approach, and also on the analytical measurement procedures used to measure the components [36,37]. Milk is a composite mixture of various proteins, carbohydrates, lipids, minerals, vitamins, etc., and CM is known to contain low amounts of fats, proteins, and oligosaccharides and high amounts of vitamins, minerals, and water [38,39]. Among macronutrients, according to meta-analysis and literature data, the fat composition of CM varies but is reportedly lower than that of milk from other domestic animals [37]. The fat component consists mainly of triglycerides and, importantly, very low levels of cholesterol [40,41]. Interestingly, the constituent of CM that is considered to have the greatest impact on its nutritional value and confer beneficial properties are proteins. Nevertheless, it is not entirely clear whether the beneficial effects of CM can be accredited to a particular component acting on one specific target or whether they are the result of the harmonious actions of multiple components in the system [7].

**Figure 1:**
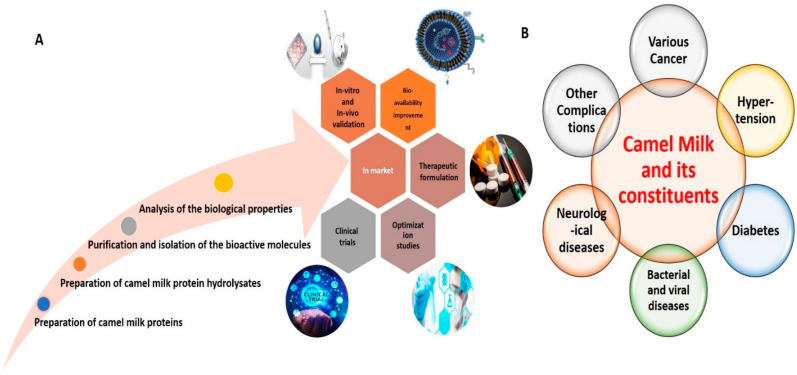
Representative image delineating various active research endeavors related to CM in food sciences (**A**). Representative image delineating the beneficial effects of CM and its constituents against various human diseases (**B**).

An overview of various active research endeavors related to CM proteins in food science is depicted in Figure 1. Basically, the main protein constituents of CM are casein proteins (CPs) and whey proteins (WPs). The proportion of CPs in CM is 50–80%, consisting of αS1-casein, αS2-casein, β-casein, and κ-casein, with an abundance of β-casein [42]. The abundance of β-casein in CM has been considered responsible for the distinctive and intriguing biological characteristics of CM, akin to those of human milk [25]. Furthermore, CPs of CM possess higher molecular weight than CPs of bovine milk (BM) [22,33,42]. In addition, the dimensions of casein micelles in CM are greater than those of casein micelles in BM [43]. WPs represent another important constituent, comprising approximately 30% of total CM proteins [44,45]. The major WPs in CM are α-lactalbumin (α-LA), camel serum albumin (CSA), lactoferrin (LF), and thermostable immunoglobulins (Igs) (IgG and IgM). In contrast to BM, wherein β-lactoglobulin (β-LG) represents the major WP component, the main WP component in CM is α-LA (50%), followed by CSA (35%) [22,25,46]. The levels of β-LG in CM are either very low or absent altogether. As β-LG is the main protein in BM responsible for eliciting allergic responses, the absence of β-LG in CM is likely responsible for its renowned anti-allergic characteristics [40,46]. Additionally, CM contains many proteins possessing immunomodulatory properties, including peptidoglycan recognition proteins (PGRPs), Igs, lactoperoxidase (LP), CSA, LF, insulin, and insulin-like proteins [37,38,47]. Among Igs, CM WPs are further reported to contain IgG1 and IgG2, IgG variants that are not present in BM. Apart from CPs and WPs, CM has some milk fat globule membrane proteins, such as milk fat globule-EGF factor 8, adipophilin, lactadherin, fatty acid synthase, and xanthine dehydrogenase. Furthermore, CM has been reported to possess high amounts of N-acetyl-β-glucosaminidase (NAGase), LP, and lysozymes (LZ) [24,48], which confer anti-bacterial, anti-fungal and anti-viral properties.

Among micronutrients, both water and fat-soluble vitamins, i.e., A, D, E, K, B complex, and C, are present in CM [39,49,50]. CM is also rich in minerals, which are present in the following order of abundance: K > Cl > Ca > P > Na > Mg, Cu, Fe, Zn, etc. [24,32,38,50,51]. It has been reported that the Fe content of CM is approximately 10-fold that in BM and that the K and Cu contents are also higher [38,50]. These elements play important roles in various biological processes, serving either as catalytic or structural components or having specific functions, all indispensable for cellular function [38,50], thereby imparting additional value to CM. In particular, the concentrations of heavy metals in CM are in the harmless range [38,50].

It is argued that most of these bioactive components, including the beneficial protein components (LF, LZ, Igs, PGRP, glutathione peroxidase, and superoxide dismutase), minerals, and vitamins, are present naturally in the raw CM; other components, such as bioactive peptides, are produced from their intact protein counterparts following digestive action by digestive enzymes or through the action of microbial enzymes during fermentation [24,52,53]. Accumulating evidence has shown that these bioactive molecules represent the pharmacologically active constituents of CM and have attracted much attention in the recent past [14,54,55,56]. On a global scale, alternative and complementary medicines have garnered great attention in biomedical research. It is widely accepted that the use of various bioactive molecules can improve pathological conditions and promote cellular homeostasis, while causing minimal side effects [57,58,59]. Accordingly, the nutraceutical and biomedical importance of CM has been the focus of many research endeavors globally, with many reviews recently compiled taking these aspects into consideration [24,30,52,60]. Representative tables delineating the therapeutic potential of CM and its constituents, especially its bioactive peptides against various diseases, have been collated, as seen in Table 1 and Table 2.

Through systematic efforts using in vitro and in vivo models, our laboratory and others have accumulated evidence on the therapeutic potential of CM components against various disease conditions [10,14,32,33,53,61,62,63,64,65,66]. Nevertheless, despite the plethora of recent studies on the pharmacological potential of protein hydrolysates (PHs) and bioactive peptides in CM, there are many stumbling blocks to leverage their full potential, seemingly due to a lack of advanced technologies, defined molecular approaches, and clinically useful formulations. Moreover, thorough animal and clinical studies are needed to fully determine the efficacy of these bioactive molecules in a true sense. The bio-availabilities of these molecules are another challenge, as these molecular entities have to be resistant toward the gastro-intestinal (GI) digestive enzymes. Thereafter, they have to be absorbable through the GI barrier in appropriate amounts so as to finally enter into the blood-stream to reach their target in pharmacologically active form to exert their activities. Reckoning with this in mind, there is a consensus that there is a need to develop strategies to utilize these hydrolysates/bioactive peptides in a commercially efficient manner. In parallel, it is also important to thoroughly assess their toxicity profile, stability, and allergenicity issues as well [61,67]. Much effort has been expended on developing various pharmacologically relevant formulations/nano-formulations aimed at revolutionizing the therapeutic regimen of CM-derived PHs and bioactive peptides [61,67,68]. Indeed, these investigations will be a challenge for the coming years; nevertheless, there are many hopes as well.

### 2.2. Pharmacological Properties of CM Bioactive Molecules against Various Pathological Conditions

#### 2.2.1. Molecular Intricacies of Anti-Cancer Effects

Cancer is a very complex disease caused by genetic and/or epigenetic changes that lead to uncontrolled cell growth. According to GLOBOCAN 2020 reports, cancer is the second leading cause of death worldwide, with an estimated 19.3 million cases diagnosed and 10.0 million deaths [91]. The report also predicts that the number of cases will nearly double by 2040 [91]. During tumorigenesis and tumor progression, cancer cells undergo several alterations, including gaining the ability to proliferate, independent of normal growth-promoting or inhibitory signals, invading and migrating to surrounding or distant tissues/sites, promoting angiogenesis, escaping apoptosis, and avoiding replicative senescence and immune responses. It has been posited that these characteristic features are acquired from alterations in cellular signaling pathways that, in normal cells, regulate controlled cell growth.

**Figure 2:**
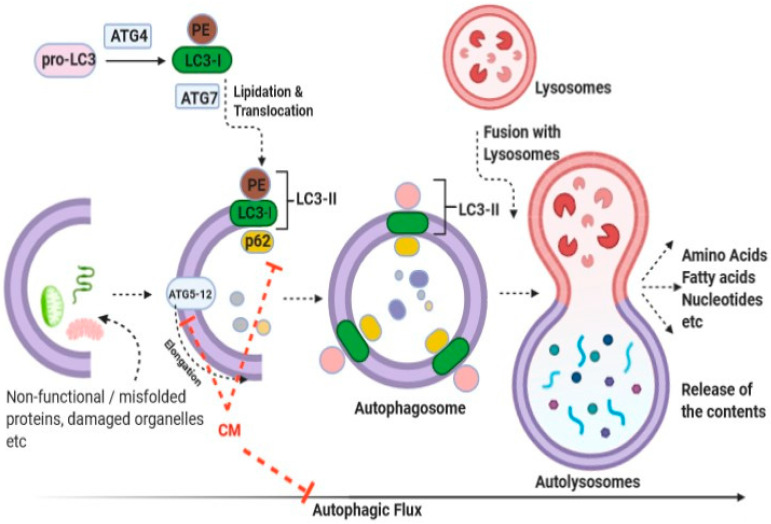
Representative image delineating the anti-cancer effect of CM against human colorectal and breast cancer cells through induction of autophagic responses (abbreviations are: LC3: a microtubule- associated protein 1 light chain 3 (LC3-I precursor); PE: phosphatidylethanolamine; LC3-II: Lipidated LC3-I; ATG4 and ATG7, autophagy proteins; ATG5-12, a complex of autophagy proteins; p62: sequestosome 1). Modified from Izadi et al., Journal of Functional food [24].

Traditionally, it was believed that the consumption of CM helped reduce the incidence of cancers; however, molecular evidence for this health benefit is limited [70,71,74]. Various in vitro studies have revealed the inhibitory effects of CM and its constituents against various forms of cancer, apparently through induction of apoptotic pathways [2,12,69,72,73,74,75,76]. In parallel, animal experimental data have also demonstrated the inhibitory potential of CM and its constituents against various forms of cancer [77,78,79]. Interestingly, CM has been shown to interfere with the expression of cancer-related mediators at both gene and protein levels [69]. Furthermore, apart from investigations into the traditional processes, reports have linked autophagic responses with the ameliorative effects of CM against cancer [71]. Autophagy, a process through which the system maintains cellular homeostasis through the removal of dysfunctional organelles and/or dysfunctional/misfolded proteins, has also been studied in relation to particular complications [92]. To this end, Krishnankutty et al. showed that CM exerts anti-proliferative effects on human colorectal cancer cells by orchestration of autophagic responses [71]. An overview of the autophagy process, together with the plausible targets of CM, is illustrated in Figure 2. Interestingly, using the GFP-LC3 puncta assay, Krishnankutty et al. clearly showed an increment in LC3-II formation following treatment with CM. Moreover, a dose-dependent decrease in p62 (sequestosome 1) was observed following treatment with CM. A dose-dependent decrease in the expression of Atg5-12 proteins was observed in two cell lines studied following treatment with CM. Collectively, the group convincingly demonstrated that CM exerted cytotoxicity responses toward human colorectal cancer cells, seemingly through orchestration of autophagic responses, although the identity of the component of CM responsible was not ascertained [71]. Further studies in this direction would be highly instrumental in identifying the active “wonder” component(s) of CM that can selectively kill cancer cells. 

Furthermore, as a matter of fact, widely used chemotherapeutic drugs have been shown to manifest many negative side effects. Nevertheless, CM has been shown to manifest many beneficial properties without any side effects. Interestingly, these studies suggest that components of CM can modulate signaling cascades and, thus, may represent an alternative to traditional chemotherapeutic drugs and/or act as adjuncts and complements in the management of various disorders, including cancer.

#### 2.2.2. Molecular Intricacies of CM’s Anti-Hypertensive Potential

Hypertension, a high blood pressure (BP) condition, leads to severe health complications and increases the risk of heart disease, stroke, and eventually death. Elevated BP causes long-term detrimental effects on the heart and other organs. In recent years, due to the increasing incidence of hypertensive heart disease, hypertension has emerged as a leading cause of cardiovascular-related morbidity and mortality worldwide [57,58,93]. Several explanations for the mechanism governing hypertension have been proposed [94,95,96]. Accumulating studies have shown that CM and CM-derived proteins and peptides have anti-hypertensive effects [12,22,33,73,82]; generally attributed to the inhibition of angiotensin-converting enzyme (ACE) [33,81,97]. To this end, we have recently performed comparative profiling of the bio-macromolecular fractions of CM and BM and ascertained their anti-hypertensive potentials following simulated gastrointestinal digestion [33], and our recent report identified novel anti-hypertensive bioactive peptides from CM protein hydrolysates (CMPHs) and delineated the underlying molecular mechanism of these peptides [10].

**Figure 3:**
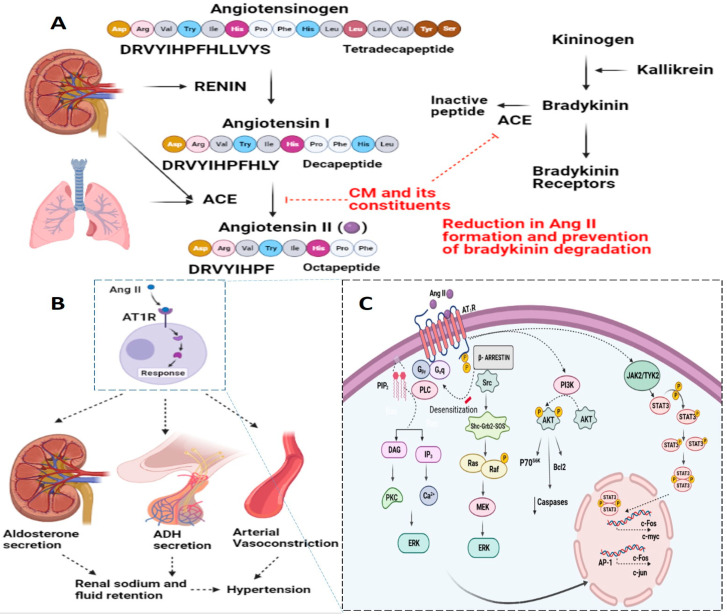
Representative image delineating the plausible anti-hypertensive effect of CM. At the molecular level, the renin-angiotensin system (RAS) is at the center of the regulation of hypertension. Angiotensin II (Ang II), an important effector RAS hormone, has been implicated in regulating the physiological effects of the regulation of blood pressure and is associated with the pathophysiology of hypertension. Basically, Ang II is derived from Ang I through the activity of Angiotensin Converting Enzyme (ACE). ACE, or kininase II, also plays a key role in the kallikrein-kinin system by cleaving bradykinin to inactive peptides; which, in turn, also affect hypertensive responses (**A**). Molecular signaling through AT_1_R is envisaged to mediate aldosterone secretion, ADH secretion, and the arterial vasoconstriction, which ultimately leads to hypertensive response (**B**). A detailed overview of the molecular signaling mediated by Ang II-AT1R is delineated (**C**). Interestingly, CM and its constituents have been reported to inhibit ACE.

The renin–angiotensin system (RAS) is a principal regulatory hormonal system that plays important roles in hypertension [57,58,96,98]. The important effector RAS hormone angiotensin II (Ang II), a vasoconstrictive peptide, has been implicated in regulating the physiological effects of the regulation of BP and is associated with the pathophysiology of hypertension [96,98,99]. Ang II has also been associated with inflammatory responses, endothelial dysfunction, atherosclerosis, and congestive heart failure. Reports have indicated various effects of Ang II, depending on the cell/tissue type under consideration and the duration of exposure (acute versus chronic) [73,96,100]. Ang II is derived from Ang I through the activity of ACE [101,102]. ACE, or kininase II, also plays a key role in the kallikrein-kinin system by cleaving bradykinin to inactive peptides; which, in turn, also have effects on hypertensive responses (Figure 3). Ang II receptors are categorized into two types based on their structure: Ang II type 1 receptor (AT_1_R) and Ang II type 2 receptor (AT_2_R), each with a distinctive downstream signaling cascade [101,102]. Signaling through AT_1_R is envisaged to mediate vasoconstriction, aldosterone secretion, catecholamine release, and cardiac remodeling [103]. However, signaling through AT_2_R has opposite effects to those mediated by AT_1_R and has been shown to induce vasodilation [104] and attenuation of cardiac remodeling [105,106].

Ang II has been shown to mediate downstream signaling through the action of G protein- and non-G protein-related signaling mediators [96,98]. However, Ang II also mediates its functions via various mitogen-activated protein kinases, receptor tyrosine kinases and non-receptor tyrosine kinases [98]. In addition, Ang II-AT_1_R-mediated NAD(P)H oxidase activation, which has been widely implicated in vascular inflammatory and fibrotic responses, has been studied in detail. These signaling pathways regulate normal cellular function and/or disease conditions. A detailed overview of the signal transduction pathways related to Ang II-mediated hypertensive responses is delineated in Figure 3. The plausible target for CM and its constituents has also been highlighted (Figure 3).

Interestingly, the anti-hypertensive potential of CM proteins, PHs and/or bioactive peptides is an important emerging area with promising prospects. It is important to understand how these CM bioactive molecules influence the cellular system at the molecular, cellular, and organelle levels. Further, it is anticipated that the underlying events do not function in isolation but rather influence each other either directly or indirectly, and ultimately affect the underlying responses. Thus, it is equally imperative to understand how these CM bioactive molecules influence these dynamically intertwined responses.

#### 2.2.3. Molecular Intricacies of CM’s Anti-Diabetic Potential

Diabetes mellitus (DM) is a group of metabolic disorders characterized by a chronic hyperglycemic condition resulting from defects in insulin secretion, insulin action, or both. The prevalence of diabetes is escalating rapidly, and the WHO has predicted that the number of adults with diabetes will have almost doubled by 2030 [107,108]. Accumulating evidence has highlighted the anti-diabetic potential of CM. To this end, a trial study by Agrawal et al. have shown that consumption of CM led to zero prevalence of diabetes in a community consuming CM regularly, compared to other communities [16]. Beside this, Alkurd et al. have highlighted the effect of CM on glucose homeostasis in diabetic patients through a systematic review and meta-analysis of randomized controlled trials [11,109].

Nevertheless, as of yet, the underlying molecular intricacies related to their beneficial effects are poorly understood [7,14,53,63,109,110]. Although various studies have offered plausible mechanistic insights, the issue still remains contentious [7,15,111,112]. It has been proposed that the presence of high levels of insulin, or insulin-like molecular entities, in CM, the protective effect of small-sized Igs in CM on pancreatic β-cells, and the absence of CM coagulation in the gastrointestinal tract, all contribute to CM’s anti-diabetic potential [34,113]. Nevertheless, these aspects have undergone a paradigm shift as a consequence of independent studies that have explicitly highlighted that these insulin/insulin-like peptides are completely hydrolyzed by the gastrointestinal enzymes [19,20,21] This has prompted the researcher to study the CM protein hydrolysates and their bioactive peptides; indeed, lately this area of research has garnered much attention all across the globe [5,22,23,24].

As a matter of fact, the pathophysiology related to diabetes is inherently complex, and it has been argued that numerous complex changes lead to a pathological condition that eventually affects the system in a multifaceted manner. To this end, efforts at our laboratory have been driven by the need to understand the molecular and cellular basis of the anti-diabetic potential of CM [7,14,15,53,54,62]. Recently, we identified positive bioactive peptides from CMWPH demonstrating dual action on ACE in vitro and on the insulin receptor in cell lines [14]. Interestingly, CM peptides showed positive allosteric modulation of the insulin receptor, and some inhibited ACE activity in vitro. Direct modulation of insulin receptor activity and its downstream signaling pathways, such as ERK1/2 and Akt, has been demonstrated [14,15]. This not only lends further support to the anti-diabetic effects of CM but also unlocks promising avenues of investigation toward identifying the CM bioactive agent and its potential application in the management of diabetes. Other bioactive peptides from CMWPH with positive effects against α-amylase and α-glucosidase have been reported by Baba et al., 2021 [24]. The inhibitory effects of CMWPH against dipeptidyl peptidase IV (DPP-IV) and inflammation have also been highlighted [62]. Similarly, Nongonierma et al. have delineated the efficacy of novel peptides from CMPH, CMWPH, and CMWP-enriched hydrolysate against DPP-IV inhibition [64,65]. In fact, human trials have also highlighted the anti-diabetic potential of CM [11,16,17,18,114,115,116]. Thus, we and others have highlighted the potential implication of CMW protein/peptides in the anti-diabetic properties of CM. In the same line, one of our recent studies found that besides these CMW protein/peptides, LF represents another potential candidate in CM. The data of the study convincingly explains that it might be one of the prospective wonder anti-diabetic agents responsible for the anti-diabetic potential of CM [6]. Collectively, all these studies may constitute a substantial advancement toward the identification of the most prospective anti-diabetic agent contained in CM. 

**Figure 4:**
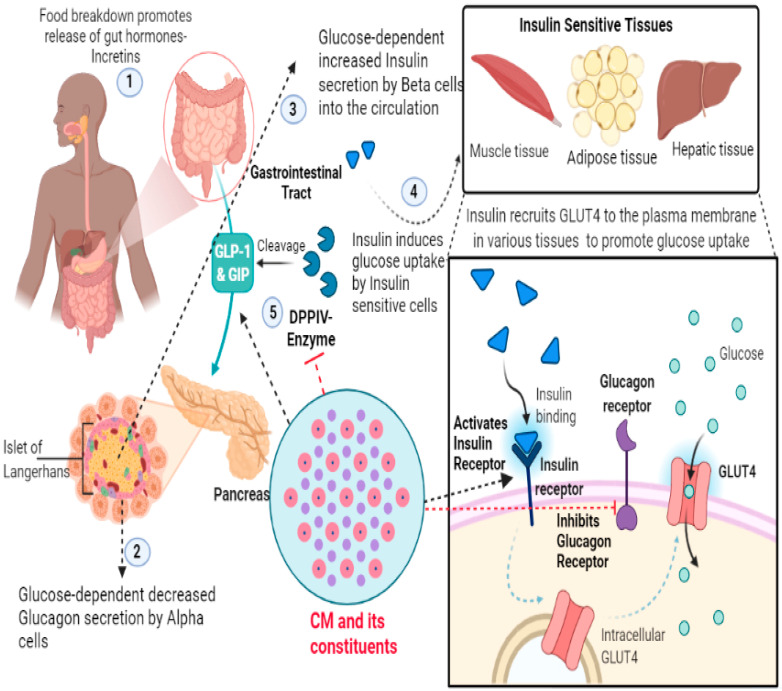
Representative image delineating the plausible anti-diabetic effect of CM. Food breakdown in the gastrointestinal tract (GI) leads to release of gut hormones, such as Glucagon-Like Peptide-1 (GLP-1) and Glucose Dependent Insulinotropic Polypeptide (GIP), which seemingly stimulate glucose-dependent insulin secretion by the pancreatic Beta cells. Insulin thereby promotes glucose uptake by the insulin sensitive tissues. Mechanistically, insulin, upon binding to insulin receptors, initiates a signaling cascade that eventually induces translocation of glucose receptors (GLUTs) to the membrane whereby glucose can be up-taken. These gut hormones are cleaved by DPP-IV enzymes which leads to attenuation of insulin secretion. Interestingly, CM and its constituents have been reported to activate GLP1/GIP and inhibit Dipeptidyl peptidase-IV (DPP-IV), activate insulin receptor and inhibit glucagon receptor. Additionally, it has been reported that CM embodies insulin-like peptides that mimic insulin responses, another aspect adding to their anti-diabetic potential.

In the recent past, research delineating the anti-diabetic properties of CM has revealed that these CM components target many key intracellular signaling pathways, especially those involved in insulin function and/or glucose homeostasis [6,14,15,53]. To this end, there have been many perspectives put forward to explain the anti-diabetic effects and other beneficial potentials of CM and its constituents [11,18,114,115,117,118,119]. In the same line, our previous review, entitled “*The molecular basis of the anti-diabetic properties of camel milk”* provides a better understanding of the anti-diabetic effects of CM proteins/peptides [7]. Taken together, all these studies provide a sound basis to better understand the putative underlying mechanism for their anti-diabetic potential. A representative image delineating the plausible anti-diabetic effect of CM and its constituents has been provided in the Figure 4.

#### 2.2.4. Molecular Intricacies of CM’s Anti-Microbial Potential

Infectious diseases are one of the leading causes of mortality and morbidity worldwide [120]. CM possesses intriguing anti-microbial potential. including antibacterial properties against both gram-negative and gram-positive bacteria [9,13,25,26,27,28,29]. Studies have shown CM’s inhibitory effects against various bacterial strains, including gram-positive strains, such as *Staphylococcus aureus, clostridium, Listeria monocytogenes, Bacillus cereus,* and gram-negative strains, including *Escherichia coli, Klebsiella pneumoniae, Salmonella typhimurium, and Helicobacter pylori* etc. [27,52,83]. Besides these effects, CM possesses antiviral properties against hepatitis C virus, cytomegalo virus, rotavirus, herpes simplex virus-1 and human immunodeficiency virus [121]. Moreover, they have been reported to possess antifungal properties (*Candida albicans*); although much less literature is available in this regard [84,122]. It has been argued that these effects were mainly attributed to the presence of greater amounts of LF, LZ, Igs, NAGase, PGRPs and LP etc. [9,25]. It is reasonable to argue that, although limited reports are available on CM proteins and their hydrolysates for their anti-microbial activities, the results are promising [26]. There is general consensus that more extensive studies exploring the anti-microbial potentials of CM protein hydrolysates and bioactive molecules against a wide range of pathogenic microorganisms in vitro as well as in vivo are of vital and immediate importance.

### 2.3. Non-Nutritional Components (CM Exosomes)

While the nutritional components of CM have long been recognized and studied in depth, research on the non-nutritional components of CM has recently accelerated [1,3]. Since antiquity, milk, especially CM, has been shown to possess various desirable pharmacological properties [1,15,52,110]. As already mentioned, accumulating evidence has shown that CM bioactive molecules possess various beneficial characteristics, including anti-oxidant, anti-microbial, anti-radical, anti-cancer, anti-hypertension, anti-diabetic, anti-inflammatory, anti-allergic, anti-autism, immunomodulatory effects, etc. [7,15,24,63]. Recent studies have highlighted that these properties can be attributed to the presence of EVs, especially exosomes [1,3]. Milk exosomes have attracted much attention, primarily due to their intrinsic beneficial properties but also because they can serve as nanodrug delivery platforms for therapeutic agents and molecular entities. Further fabrication of exosomes with targeting moieties would allow targeted delivery of drugs/molecular entities to the desired sites.

Exosomes are naturally occurring, nano-sized (20–100 nm) EVs that are released from almost every cell type [123,124]. They are found throughout the body in the extracellular environment and biofluids, including cerebrospinal fluid, serum, saliva, urine, and milk [123,124,125,126,127]. They are mechanistically and functionally diverse from their cellular counterparts and are more heterogeneous, depending on their origin. They are lipid bilayer assemblies containing membrane-bound and internal proteins and a wide range of nucleic acid moieties [124]. It is thought that cellular communication by exosomes occurs through their ability to shuttle cargoes of proteins, circulating DNA, noncoding RNAs, lipid moieties, and metabolites into their surrounding milieu [124,128,129,130]. For example, it has been shown that milk exosomes shuttle genetic material from parents to offspring, thereby playing a role in the regulation of the infant’s development [129].

## 3. Pharmacological Properties of CM Exosomes

Evidence has shown that exosomes derived from human milk, BM, and CM exhibit antitumor activities [35,131]; however, reports on their anti-diabetic and anti-hypertensive potential are scarce.

### 3.1. Molecular Intricacies of Their Anti-Cancer Potential

There are many reports on the anti-cancer potential of milk-derived exosomes from bovine sources, and research on CM exosomes is still in its infancy. To the best of our knowledge, Badawy et al. were the first to evaluate the potential effects of CM and its exosomes against breast cancer; they evaluated their chemotherapeutic potential and provided evidence for the underlying mechanism of action [1]. They suggested that the anti-cancer effect of CM exosomes was mediated through the regulation of apoptosis and suppression of angiogenesis, metastasis, and inflammatory responses and that these effects were attributable to miRNA constituents of the exosomes [1]. CM exosomes have also been reported to mitigate oxidative stress and immuno-toxic responses induced by the chemotherapeutic drug cyclophosphamide in an albino rat model [3]. Recently, El-Magd have envisaged the apoptotic, anti-inflammatory, and anti-angiogenesis properties of CM exosomes from colostrum against HepaRG cells [80].

### 3.2. CM Exosomes as a Natural Biogenic Nano-Delivery Platform for Therapeutics

Milk-derived exosomes have garnered much interest, not only for their intrinsic beneficial properties, but also for their ability to serve as biomimetic nanodrug delivery platforms. The use of milk exosomes as a delivery vehicle for therapeutic drugs and/or siRNA, and the development of exosome-based targeted delivery platforms, constitute an emerging and exciting field.

Gupta et al. were the first to examine the potential of bovine exosomes as a biogenic nano-delivery platform for chemotherapeutic drugs, such as paclitaxel, curcumin, anthocyanidins, celastrol, and siRNA [132,133,134,135,136]. Yassin et al. described a method for isolating CM exosomes using differential ultracentrifugation and provided an in-depth characterization of CM exosomes [66]. They showed that CM exosomes have a truncated 35-kDa protein of the TSG101 marker (compared with the mammalian 43-kDa protein) and an average size of approximately 30 nm. Additionally, using phospholipidomic analysis, PC was found to be the most abundant phospholipid. A detailed analysis of EVs isolated from CM using liquid chromatography with tandem mass spectrometry revealed a variety of protein signatures associated with small EVs, including ADAM10, TSG101, CD3, CD63, CD81, HSP70, and HSP90, which suggests that CM EVs are rich in exosomal proteins. While CM exosomes offer exciting research potential, to leverage their full potential, further standardization and optimization of isolation protocols and improved quality control are important aspects that must not be overlooked. It is equally important to assess the individual components of exosomes responsible for the underlying effects, in parallel with the assessment of their potential toxicity issues.

## 4. Conclusions

CM is widely regarded to possess extraordinary medicinal properties, including anti-cancer, anti-diabetes, anti-hypertension, anti-inflammatory, anti-allergic effects and so on. This review is expected to stimulate interest in CM research and broaden interest in the development of modern CM-based therapeutic interventions with the potential to revolutionize CM-based therapeutic regimes against a myriad of diseases. Previously, most of the beneficial properties of CM have been demonstrated for intact CM proteins. Nevertheless, in recent time, research has mainly focused on generating bioactive hydrolysates from CM proteins and exploring their potential beneficial effects. Given the ongoing research drive to elucidate the therapeutic benefits of CM, functional and nutraceutical products derived from CM may imminently be commercially available, which would definitely aid in improving worldwide health status. Furthermore, in order to broaden our understanding of the underlying molecular mechanism for the traditionally acclaimed health benefits of CM; a more practical approach may involve systems and biology and bio-informatics approaches to pinpoint signal hubs, molecular mediators and cross-roads that are common to all of the molecular signaling pathways. Certainly, the use of sophisticated animal models, multi-model co-culture systems, and novel adaptive experimental trial designs will greatly enhance CM-based therapeutic research efforts. Indeed, all these studies could provide a foundation for rationally designed, molecularly targeted, CM-derived bioactive molecule-based therapeutic interventions. 

## Figures and Tables

**Table 1:** Representative table delineating the therapeutic potential of Camel Milk and its constituents.

S.No	Camel Milk Constituents	Cell Line/Animal Model/Assay Used	Mechanism	Reference
	Cancer			
	*Nutritional Components*			
1	Camel Milk	Human Hepatoma Cell Line (Hep-G2) and Human Breast Cancer Cell Line (MCF-7)	Induction of Apoptosis	[69]
2	Camel Milk	Murine Hepatoma Hepa 1c1c7 Cell Line	Modulation of the expression of cancer-related genes (Cyp1a1), (Nqo1), and (Gsta1) at the transcriptional and post-transcriptional levels	[70]
3	Camel Milk	Human Colorectal Cancer Cell line (HCT-116) and Breast Cancer Cell Line (MCF-7)	Induction of Autophagic Cell Death	[71]
4	Lyophilised Camel Milk	Human Breast Cancer Cell Line (BT-474)	Induction of Apoptosis	[72]
5	Camel Milk Lactoferrin	Human Colon Cancer Cell Line (HCT-116)	Inhibition of Human colorectal cancer cell line (HCT-116) proliferation and DNA damage inhibitory activities	[2]
6	Camel Milk fermented with Camel Milk probiotic strain Lactococcus lactis KX881782 (Lc.K782) and control Lactobacillus acidophilus DSM9126 (La.DSM)	Human Colorectal Adenocarcinoma Cell Line (Caco-2), Human Breast Cancer Cell Line (MCF-7), and and Human Adenocarcinoma Cell Line (HELA)	Inhibition of proliferation	[12]
7	Camel Milk fermented with Camel Milk probiotic strains Lb. reuteri-KX881777, Lb. plantarum-KX881772, Lb. plantarum-KX881779 and a control strain Lb. plantarum DSM2468	Human Colorectal Adenocarcinoma Cell Line (Caco-2), Human Breast Cancer Cell Line (MCF-7) and Human Adenocarcinoma Cell Line (HELA)	Inhibition of proliferation	[73]
8	Camel Milk, Casein and Whey Proteins	Human Breast Cancer Cell Line (MCF-7)	Inhibition of proliferation as evident through MTT assay	[74]
9	Camel Milk and Whey proteins	Human Adenocarcinoma Cell Line (HELA)	Inhibition of proliferation as evident through MTT assay	[75]
10	TR35-An active fraction from Xinjiang Bactrian Camel Whey	In vitro Human Esophageal Carcinoma Cell Line (Eca-109) In vivo BALB/c nude mice subcutaneously injected with 2 × 10^6^ Eca-109 cells	Inhibition of Eca-109 cell proliferation and induction of apoptosis	[76]
11	Camel Milk	Induced diethylnitrosamine and phenobarbitone Hepatic Cancer Wistar Rat Model	Potent Inhibitory effect on hepatocarcinogenesis in Wistar Rats was observed	[77]
12	Camel Milk Whey Protein	Induced Azoxymethane (AOM)/Dextran sodium sulfate (DSS) Mouse Model	Inhibition of inflammatory colorectal cancer development via down-regulation of pro-inflammatory cytokines	[78]
13	Camel Milk	Sponge implant angiogenesis Male Swiss Albino Mice Model	Inhibition of inflammatory angiogenesis via down-regulation of pro-angiogenic and pro-inflammatory cytokines	[79]
	***Non-Nutritional Components***			
14	Camel Milk Exosomes	Human Colorectal Cancer Cell Line(HCT-116) and Human Breast Cancer Cell Line (MCF-7)	Induction of Autophagy	[1]
15	Camel Milk Exosomes	Albino Rat Model	Mitigation of oxidative stress and immune-toxic responses induced by the chemotherapeutic drug viz. cyclophosphamide (CTX)	[3]
16	Camel Milk Exosomes	HepaRG cells	Potential apoptotic, anti-inflammatory, and anti-angiogenesis effects against HepaRG cells	[80]
	**Hypertension**			
1	Camel Milk Protein and Lipid fractions	Colorimetry based analytical technique	Inhibition of Angiotensin-1 converting enzyme (ACE)	[33]
2	Bioactive Peptides from Camel Milk Protein Hydrolysates	Colorimetry based analytical technique	Inhibition of Angiotensin-1 converting enzyme (ACE) and anti-inflammatory responses	[10]
3	Bioactive Peptides from Camel Milk Casein Hydrolysates	Colorimetry based analytical technique	Inhibition of Angiotensin-1 converting enzyme (ACE) and radical scavenging activities	[81]
4	Camel Milk fermented with Camel Milk probiotic strain Lactococcus lactis KX881782 (Lc.K782) and control Lactobacillus acidophilus DSM9126 (La.DSM)	Colorimetry based analytical technique	Inhibition of Angiotensin-1 converting enzyme (ACE)	[12]
5	Camel Milk fermented with Camel Milk probiotic strains Lb. reuteri-KX881777, Lb. plantarum-KX881772, Lb. plantarum-KX881779 and a control strain Lb. plantarum DSM2468	Colorimetry based analytical technique	Inhibition of Angiotensin-1 converting enzyme (ACE)	[73]
6	Fermented Skim Camel Milk	Spontaneously Hypertensive Rats	Attenuation of systolic and diastolic blood pressure, Inhibition of Angiotensin-1 converting enzyme (ACE)	[82]
	**Diabetes**			
1	Camel Milk and Protein Fractions	Human Embryonic Kidney Cell Line (HEK-293)	Allosteric effect on insulin receptor conformation and activation; and modulation of downstream signalling	[15]
2	Camel Milk Whey Protein and Camel Milk Whey Protein Hydrolysates	Human Liver Cancer Cell Line (Hep-G2) and Human Embryonic Kidney Cell Line (HEK-293)	Inhibition of Dipeptidyl peptidase-IV (DPP-IV), Activation of insulin receptor and Positive Regulation on Glucose Uptake	[14]
3	Camel Milk Protein Hydrolysates	Colorimetry based analytical technique	Inhibition of α-amylase	[63]
4	Camel Milk Whey Protein Hydrolysates	Colorimetry based analytical technique	Inhibition of α-amylase and α-glucosidase	[54]
5	Camel Whey Protein Hydrolysates	Colorimetry based analytical technique	Inhibition of Dipeptidyl peptidase-IV (DPP-IV) and inflammation	[62]
6	Camel Milk Protein Hydrolysates	Colorimetry based analytical technique	Inhibition of Dipeptidyl peptidase-IV (DPP-IV)	[65]
7	Camel Milk Protein Hydrolysates	Colorimetry based analytical technique	Inhibition of Dipeptidyl peptidase-IV (DPP-IV)	[64]
8	Camel Whey Protein Enriched Hydrolysates	Colorimetry based analytical technique	Inhibition of Dipeptidyl peptidase-IV (DPP-IV)	[64]
9	Camel Milk Protein Hydrolysates	Streptozotocin (STZ)-induced Diabetic Rats	Potent Hypoglycemic activity, as evident by reduction in fasting Blood Glucose and Oral glucose tolerance test (OGTT) levels; Preservation of β-cells was also observed	[53]
10	Camel Milk Protein Lactoferrin	HEK-293 and Hep-G2 cells	Modulation of Insulin Receptor and downstream signalling	[6]
**Anti-microbial**				
1	Camel Milk Casein Protein hydrolysates and its fraction	*Gram positive bacteria: Staphylococcus aureus, Bacillus cereus* and *Listeria monocytogenes* *Gram negative bacteria: Escherichia coli*	Significant anti-microbial activity was observed against all the microbial strain tested for all the fractions	[27]
2	Camel Whey Proteins and hydrolysates	*Gram negative bacteria: Escherichia coli Dh1α*	Improved anti-microbial activities of Camel Whey Proteins were observed, particularly for limited Proteolysed fractions	[28]
3	Lysozyme(LZ), lactoferrin(LF), lactoperoxidase(LP), immunoglobulin G (IgG) and secretory immunoglobulin A(Ig A) extracted from camel milk	*Gram positive bacteria: Lactococcus lactis subsp. cremoris* *Gram negative bacteria: Escherichia coli, Staphylococcus aureus, Salmonella typhimurium* *Virus: Rotavirus*	Camel milk LF showed intriguing antibacterial activity. The camel milk LP was bacteriostatic against the Gram-positive strains and was bactericidal against Gram-negative cultures. The immunoglobulins had little effect against the bacteria but high titres of antibodies against rotavirus were found in camel milk. The LP system was ineffective against rotavirus	[29]
4	Camel Casein Proteins and hydrolysates	*Gram positive bacteria: Listeria* *innocua, Bacillus cereus, and Staphylococcus aureus* *Gram-negative bacteria: Escherichia coli XL1 bleu and Pseudomonas aeruginosa*	Camel milk casein hydrolysates exhibited anti-bacterial activity; Gram-positive strain growth was not affected by intact camel casein fraction, whereas the respective hydrolysates slightly inhibited the growth of the bacteria	[83]
5	Camel and Cow Casein Proteins and hydrolysates	*Candida krusei, Candida parapsilosis*	Camel milk protein hydrolysates were more potent in inhibiting pathogenic Candida species compared with cow milk protein hydrolysates	[84]

**Table 2:** Representative table highlighting some of the putative bioactive peptides from Camel Milk.

S.No	Bioactive Peptide	Mechanism	Reference
Hypertension			
1	AIPPKKNQD	Inhibition of Angiotensin-1 converting enzyme (ACE)	[85]
2	DLENLHLPLPL; LTDLENLHLPLPL;TDLENLHLPLP; TDLENLHLPLPL; TLTDLENLHLPLPL	Inhibition of Angiotensin-1 converting enzyme (ACE)	[86]
3	LSLSQFKVLPVPQ; KVLPVPQQMVPYPQ;TDLENLHLPLPL	Inhibition of Angiotensin-1 converting enzyme (ACE)	[87]
4	AEWLHDWKL; SHSPLAGFR; LTMPQWW; CLSPLQMR and CLSPLQFR	Inhibition of Angiotensin-1 converting enzyme (ACE)	[10]
5	QSAPGNEAIPP	Inhibition of Angiotensin-1 converting enzyme (ACE)	[88]
6	MVPYPQR	Inhibition of Angiotensin-1 converting enzyme (ACE)	[89]
**Diabetes**			
1	FLQY; FQLGASPY; ILDKEGIDY; ILELA; LLQLEAIR; LPVP; LQALHQGQIV; MPVQA; and SPVVPF	Inhibition of Dipeptidyl peptidase-IV (DPP-IV)	[90]
2	VPV, YPI and VPF	Inhibition of Dipeptidyl peptidase-IV (DPP-IV)	[64]
3	DNLMPQFM and WNWGWLLWQL	Inhibition of Dipeptidyl peptidase-IV (DPP-IV)	[63]
4	INNQFLPYPYWL and IPAVF	Inhibition of Dipeptidyl peptidase-IV (DPP-IV)	[65]

## Abbreviations

CM	Camel milk
CP	Casein protein
WP	whey protein
α-LA	α-Lactalbumin
CSA	camel serum al-bumin
LF	lactoferrin
ACE	Angiotensin converting Enzyme
Ang II	Angiotensin II
CMPH	CM protein hydrolysates
RAS	Renin Angiotensin system
